# Gene regulation and signaling pathways in immune response to respiratory sensitizers: a database analysis

**DOI:** 10.3389/fimmu.2025.1470602

**Published:** 2025-03-03

**Authors:** Taylor Jefferis, James Y. Liu, Kiera L. Griffin, Matthew Gibb, Christie M. Sayes

**Affiliations:** ^1^ Department of Environmental Science, Baylor University, Waco, TX, United States; ^2^ Institute of Biomedical Studies, Baylor University, Waco, TX, United States

**Keywords:** KEGG, GO, immune, immunoregulation, pathway analysis

## Abstract

**Introduction:**

Humans are regularly exposed to environmental substances through inhaled air. Some chemicals or particles are respiratory sensitizers that can cause adverse respiratory health effects by triggering amplified immune responses. Understanding the process of respiratory sensitization and identifying potential sensitizers have been challenging due to the complexity of the underlying mechanisms.

**Methods:**

This study leverages the transcriptomics from a previous *in vitro* 3D human lung model to investigate the pathways of chemical respiratory sensitization. Differentially expressed genes between two known and two nonsensitizers are cross-referenced against databases on biological processes and disease pathways.

**Results:**

The GO results revealed 43 upregulated genes, and the KEGG revealed 52. However, only 18 upregulated genes were common between GO and KEGG. The GO results revealed 26 downregulated genes, and the KEGG revealed 40. However, only 9 of those downregulated genes were common.

**Discussion:**

These findings support using multiple databases in perturbed gene analyses. The results from this study and data available in the scientific literature contribute toward building a biomarker profile for identifying respiratory sensitizers.

## Introduction

The main objective of this paper is to emphasize the benefits of using multiple databases in pathway analysis to determine the potential of a chemical to act as a respiratory sensitizer. RNAseq generates an overwhelming number of genes that are either upregulated or downregulated. RNAseq data must be processed systematically to ensure reliable and rigorous interpretation. We propose utilizing online, free, publicly available bioinformatics database tools to reduce the millions of genes produced from sequencing to a manageable number that can generate testable hypotheses in an *in vivo* study or clinical trial. Therefore, we have demonstrated another valid reason for conducting *in vitro* screening tests to complement the previously established reasons for conducting *in vitro* and *in vivo* testing (1, 2).

Through inhalation, people can be exposed to various exogenous materials found in the air, including chemical vapors, particulate matter, and microbes that may cause acute or chronic lung irritation (2–6). In addition, people can be exposed to asthmagens, which can lead to hyperreactivity symptoms without an immunological reaction (7–11), whereas inhalation of respiratory sensitizers may cause long-term adverse immunological outcomes in the lungs due to amplified responses from repeated exposures (12).

In a previous study, researchers investigated the transcriptome of dendritic cells, macrophages, and epithelial cells (2). After examining these transcriptomes of cells exposed to two known respiratory sensitizers and comparing them to cells exposed to two known non-sensitizers, a better idea of the effects of several different lung cells can be achieved. Compared to a less representative mono-culture model, the multi-cell culture system helps to mimic the relative lung compartment’s effects. This data was then used for an in-depth pathway analysis as a low-cost alternative to traditional *in vivo* models (13).

Pathway analysis combines biomolecular function knowledge with statistical techniques to interpret high-complexity biological data (14–16). However, these analyses are not routinely reported upon in the literature. Measuring multiple genes in a single-cell system enables advanced synthesis into possible perturbed biological processes. To that end, databases such as the Alliance of Genome Resources Gene Ontology (GO) Consortium and the Kyoto Encyclopedia of Genes and Genomes (KEGG) can elucidate pathways associated with the transcriptome of respiratory sensitizers (17, 18). GO provides consistent descriptors for gene products and standardizes classifications for sequences and their features (19–22).

Investigating novel methods for identifying respiratory sensitizers will ultimately improve health and safety outcomes for those exposed to these materials. Our findings will contribute to building a biomarker profile to identify respiratory sensitizers using pathway analysis. However, the main objective of this paper is to emphasize the benefit of using multiple databases in pathway analysis to conclude the ability of a chemical to be a respiratory sensitizer. It is hypothesized that using multiple databases will identify more perturbed genes that may arise from exposure to respiratory sensitizers than previously discovered.

## Materials and methods

### Experimental overview

Part of the methods for this paper are adapted from the previously mentioned study (2, 23–27). The only difference in methods from the previous paper to this one is the addition of analysis using KEGG and GO together rather than GO by itself. This allows for a more comprehensive analysis of the RNAseq data from the previous study.

### The multiple cell culture model system

A multi-cell culture system was developed using epithelial cells (A549), macrophages (differentiated U937), and dendritic cells (differentiated THP-1) in a 12-well plate fitted with polyethylene terephthalate (PET) Transwell^®^ membranes (Corning, Tewksbury, MA, USA). Cells were cultured in complete RPMI (cRPMI) 1640 (Thermo Fisher Scientific Inc. Waltham, MA, USA) with 10% FBS and 1% penicillin-streptomycin. The dendritic cells were also supplemented with 2-mercaptoethanol at a final concentration of 0.05 mM. All cells were maintained at 37°C in a humidified 5% and CO_2_ atmosphere.

Monocytes (U937) were incubated for 24 hr with 100 ng/mL phorbol 12-myristate-13-acetate (PMA) to differentiate into macrophages (28). The cells were washed twice with sterile 1X PBS before replenishing the media and allowing them to rest in the incubator for 72 hours before trypsinization and resuspension to be counted and plated. Monocytes (THP-1) were centrifuged and resuspended at 2×10^5^ cells/mL, then cultured in a serum-free medium supplemented with rhIL-4 (200 ng/mL), rhGM-CSF (100 ng/mL), rhTNFa (20 ng/mL), and 200 ng/mL ionomycin, all purchased from Thermo Fisher Scientific. To complete differentiation into dendritic cells, they were left to rest for 48 hr in the incubator before plating.

Epithelial cells were plated with a seeding density of 28×10^4^ cells/cm^2^ and allowed to adhere for 72 hr. The inserts were then inverted and placed into sterile glass Petri dishes so that dendritic cells could be plated at a seeding density of 7×10^4^ cells/cm^2^ on the basal surface of the membrane and allowed to adhere for 4 hr. Inserts were then reverted and placed back into the well plate to seed the macrophages at a 1:9 ratio of U937:A549. The media was supplemented with 2-mercaptoethanol and added to the basolateral chamber of the wells, and the total volume was replenished to 500 μL. The model was left in the incubator for 24 hr before adding chemical exposures.

### Chemical exposures

Isophorone diisocyanate (IPDI, Thermo Fisher Scientific, Inc.) was added at 25 μM, and ethylenediamine (ED, Thermo Fisher Scientific, Inc.) was added at 500 μM as positive controls for respiratory sensitization (29–33). Chlorobenzene (CB, Thermo Fisher Scientific, Inc.) was added at 98 μM, and dimethylformamide (DF, Thermo Fisher Scientific, Inc.) at 500 μM as negative controls/non-sensitizers (29, 34). Each chemical was introduced only to the apical compartment in its respective concentration. A vehicle (dimethyl sulfoxide, DMSO) was used to solubilize IPDI and CB. After 24 hr of chemical exposure, RNA extraction and sequencing were performed at Azenta Life Sciences (South Plainfield, NJ, USA).

### RNA extraction

Total RNA was extracted from frozen pellets using RNeasy Plus Universal mini kit (Qiagen, Hilden, Germany). RNA was quantified using a Qubit 2.0 fluorometer (Life Technologies, Carlsbad, CA, USA), and the integrity was checked using Agilent TapeStation 4200 (Agilent Technologies, Palo Alto, CA, USA). Sequencing libraries were then prepared using the NEBNext Ultra II RNA Library Prep for Illumina (NEB, Ipswich, MA, USA). mRNAs were fragmented for 15 min at 94°C and enriched with Oligod(T) beads. First and second-strand cDNA was synthesized, and universal adapters were ligated to cDNA fragments, which were repaired and adenylated at 3’ ends. Then, index addition and library enrichment by PCR before the libraries were validated on the Agilent TapeStation (Agilent Technologies) and quantified by using Qubit 2.0 Fluorometer (Invitrogen) along with quantitative PCR (KAPA Biosystems, Wilmington, MA, USA).

The sequencing libraries were clustered on a lane of a HiSeq flow cell, and samples were sequenced using a 2×150 bp paired-end (PE) configuration. The software then conducted image analysis and base calling. The raw sequence data (.bcl files) generated by the sequencer were converted into fastq files and de-multiplexed using Illumina’s bcl2fastq 2.17 software. One mismatch was allowed for index sequence identification.

### Data analysis

Following sequencing, the raw data was processed for further analysis. Raw reads were checked for quality, trimmed to remove adaptor sequences, and mapped to the reference genome (ENSEMBL; STAR aligner v.2.5.2b). Unique gene hit counts were calculated using feature counts of reads in exonic regions (Subread package v.2.5.2b).

Determination of differential expression was done using the R programming language using the packages gplots, ggplot2, viridis, dplyr, tidyverse, GO.db, annotate, org.Hs.eg.db, and circlize. Log-2-fold-change (L2FC) was calculated for all treatments normalized to untreated controls and for all sensitizers normalized to non-sensitizers. Genes with L2FC values > 1 and p-values < 0.05 (Wald test) were considered as differentially expressed (35). Differentially expressed genes were then input into the GO and KEGG databases, and the impacted terms and pathways were extracted, respectively.

The top ten perturbed GO terms and KEGG pathways were reported for each compartment using chord diagrams with a hierarchical distance from the root (biological process) of five. Data for all the differentially expressed genes was then used to analyze the top ten most affected GO terms and KEGG pathways for all samples pooled together.

Differentially expressed genes were extracted from raw hit counts in R and used for pathway analysis. The most common terms for GO biological processes with a hierarchical distance of 5 from the root were collected using the differentially expressed genes grouped by the apical and basal compartments. Chord diagrams were generated in R using the *circlize* package, mapping relationships between the top ten terms and their associated genes. The process was followed using KEGG pathways, and chord diagrams were generated using the top 10 pathways.

## Results

**Figure 1:**
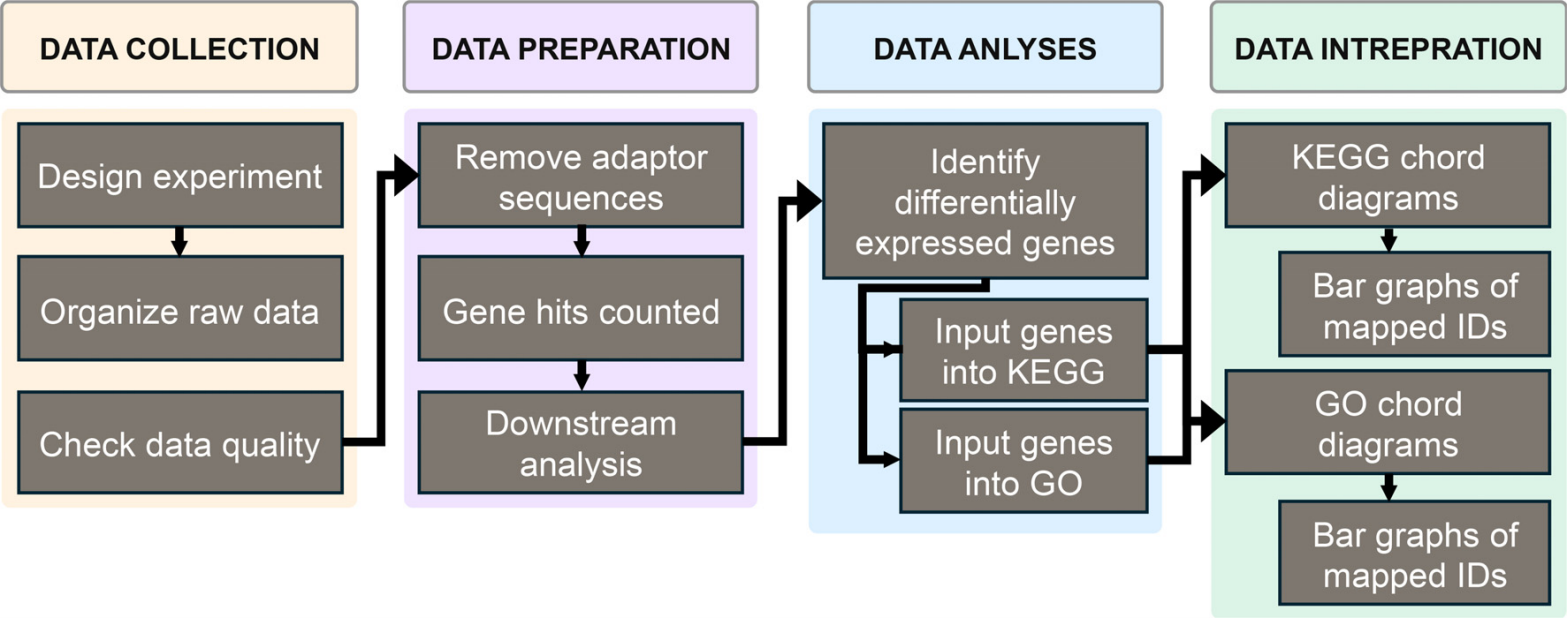
The overall methods used to obtain the data in this study.

This study utilized data collected from a previous study to expand upon the previous understanding and develop a more comprehensive understanding of the ability to use pathway analysis in distinguishing sensitizers from non-sensitizers. **Figure 1** shows an overview of the methods used to obtain this information. First, data was obtained from the previously mentioned study. Then, the differentially expressed genes from the aggregate of each compartment were entered into the GO and KEGG databases to aid in forming chord diagrams to display the results.

GO includes terms that provide unique information to group genes based on their commonalities, which helps conclude which genes are differentially expressed (29). GO is hierarchal, which splits into biological processes, cellular components, and molecular functions (30, 31). KEGG draws information about cells or organisms based on their genome information (36, 37). This is used to further information about pathway analysis and is more specific to disease pathology, similar to the mechanisms involved in the immune response to respiratory sensitization in humans (36).

**Figure 2:**
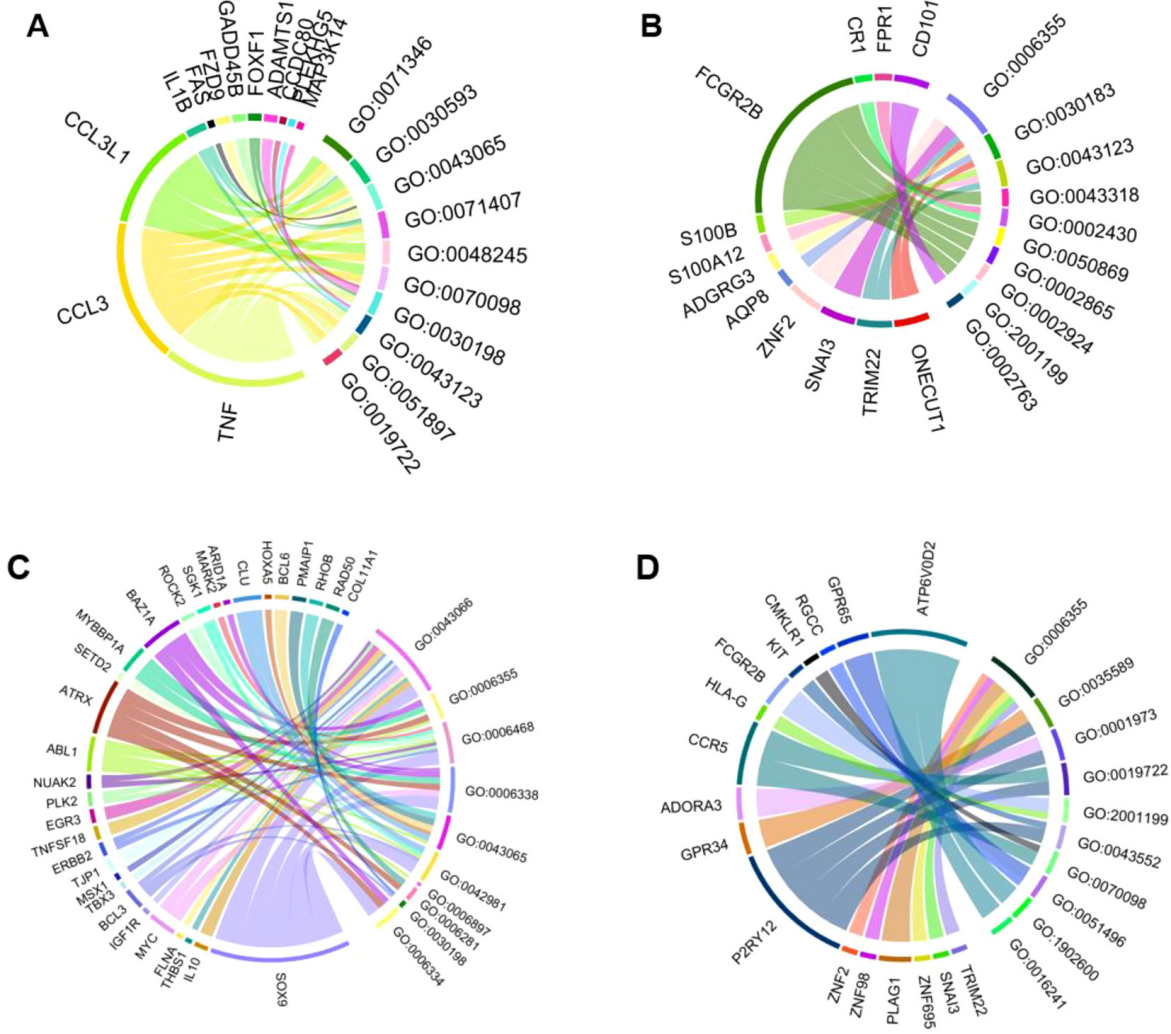
Chord diagrams depicting the associations between differentially expressed genes for cells and GO terms for biological process (minimum distance of 5 from root). **(A)** The top 10 GO terms for upregulated genes in the apical compartment. **(B)** The top 10 GO terms for downregulated genes in the apical compartment. **(C)** The top 10 GO terms for upregulated genes in the basolateral compartment. **(D)** The top 10 GO terms for downregulated genes in the basolateral compartment.

GO analysis identifies pathways that may lead to potential biomarker signatures, which can help determine potential sensitizers. We prepared chord diagrams representing the data. These depict the associations between differentially expressed genes for apical cells (**Figures 2A, B**) and basal cells (**Figures 2C, D**) and GO terms for biological processes (**Table 1**). Terms with a minimum distance of 5 from the root are used. Upregulated and downregulated genes and descriptions of each GO term are shown.

**Table 1:** Description of GO Terms.

GO Term	Description
GO:0071346	cellular response to type II interferon
GO:0030593	neutrophil chemotaxis
GO:0043065	positive regulation of apoptotic process
GO:0071407	cellular response to organic cyclic compound
GO:0048245	eosinophil chemotaxis
GO:0070098	chemokine-mediated signaling pathway
GO:0030198	extracellular matrix organization
GO:0043123	positive regulation of I-kappaB kinase/NF-kappaB signaling
GO:0051897	positive regulation of protein kinase B signaling
GO:0019722	calcium-mediated signaling
GO:0006355	regulation of DNA-templated transcription
GO:0030183	B cell differentiation
GO:0043123	positive regulation of I-kappaB kinase/NF-kappaB signaling
GO:0043318	negative regulation of cytotoxic T cell degranulation
GO:0002430	complement receptor mediated signaling pathway
GO:0050869	negative regulation of B cell activation
GO:0002865	negative regulation of acute inflammatory response to antigenic stimulus
GO:0002924	negative regulation of humoral immune response mediated by circulating immunoglobulin
GO:2001199	negative regulation of dendritic cell differentiation
GO:0002763	positive regulation of myeloid leukocyte differentiation
GO:0043066	negative regulation of apoptotic process
GO:0006355	regulation of DNA-templated transcription
GO:0006468	protein phosphorylation
GO:0006338	chromatin remodeling
GO:0043065	positive regulation of apoptotic process
GO:0042981	regulation of apoptotic process
GO:0006897	endocytosis
GO:0006281	DNA repair
GO:0030198	extracellular matrix organization
GO:0006334	nucleosome assembly
GO:0006355	regulation of DNA-templated transcription
GO:0035589	G protein-coupled purinergic nucleotide receptor signaling pathway
GO:0001973	G protein-coupled adenosine receptor signaling pathway
GO:0019722	calcium-mediated signaling
GO:2001199	negative regulation of dendritic cell differentiation
GO:0043552	positive regulation of phosphatidylinositol 3-kinase activity
GO:0070098	chemokine-mediated signaling pathway
GO:0051496	positive regulation of stress fiber assembly
GO:1902600	proton transmembrane transport
GO:0016241	regulation of macroautophagy

**Figure 2** shows *TNF*, *CCL3*, *CCL3L1*, *IL1B*, *FAS*, *FZD9*, *GADD458*, *FOXF1*, *ADAMTS1*, *CCDC80*, *MAP3K14*, and *PLEKHG5* as upregulated genes in the apical compartment. The downregulated genes of the apical compartment, according to the figure, are *CD101*, *FPR1*, *CR1*, *FCGR2B*, *S100B*, *S100A12*, *ADGRG3*, *AQP8*, *ZNF2*, *SNAI3*, *TRIM22*, *ONECUT1*.

**Figure 2** shows *COL11A1*, *RAD50*, *RHOB*, *PMAIP1*, *BCL6*, *HOXA5*, *CLU*, *ARID1A*, *MARK2*, *SGK1*, *ROCK2*, *BAZ1A*, *MYBBP1A*, *SETD2*, *ATRX*, *ABL1*, *NUAK2*, *PLK2*, *EGR3*, *TNFSF18*, *ERBB2*, *TJP1*, *MSX1*, *TBX3*, *BCL3*, *IGF1R*, *MYC*, *FLNA*, *THBS1*, *IL10*, *SOX9* as upregulated genes in the basal compartment. The downregulated genes are *ATP6V0D2*, *GPR65*, *RGCC*, *CMKLR1*, *KIT*, *FCGR2B*, *HLA-G*, *CCR5*, *ADORA3*, *GPR34*, *P2RY12*, *ZNF2*, *ZNF98*, *PLAG1*, *ZNF695*, *SNAI3*, *TRIM22*.

**Figure 3:**
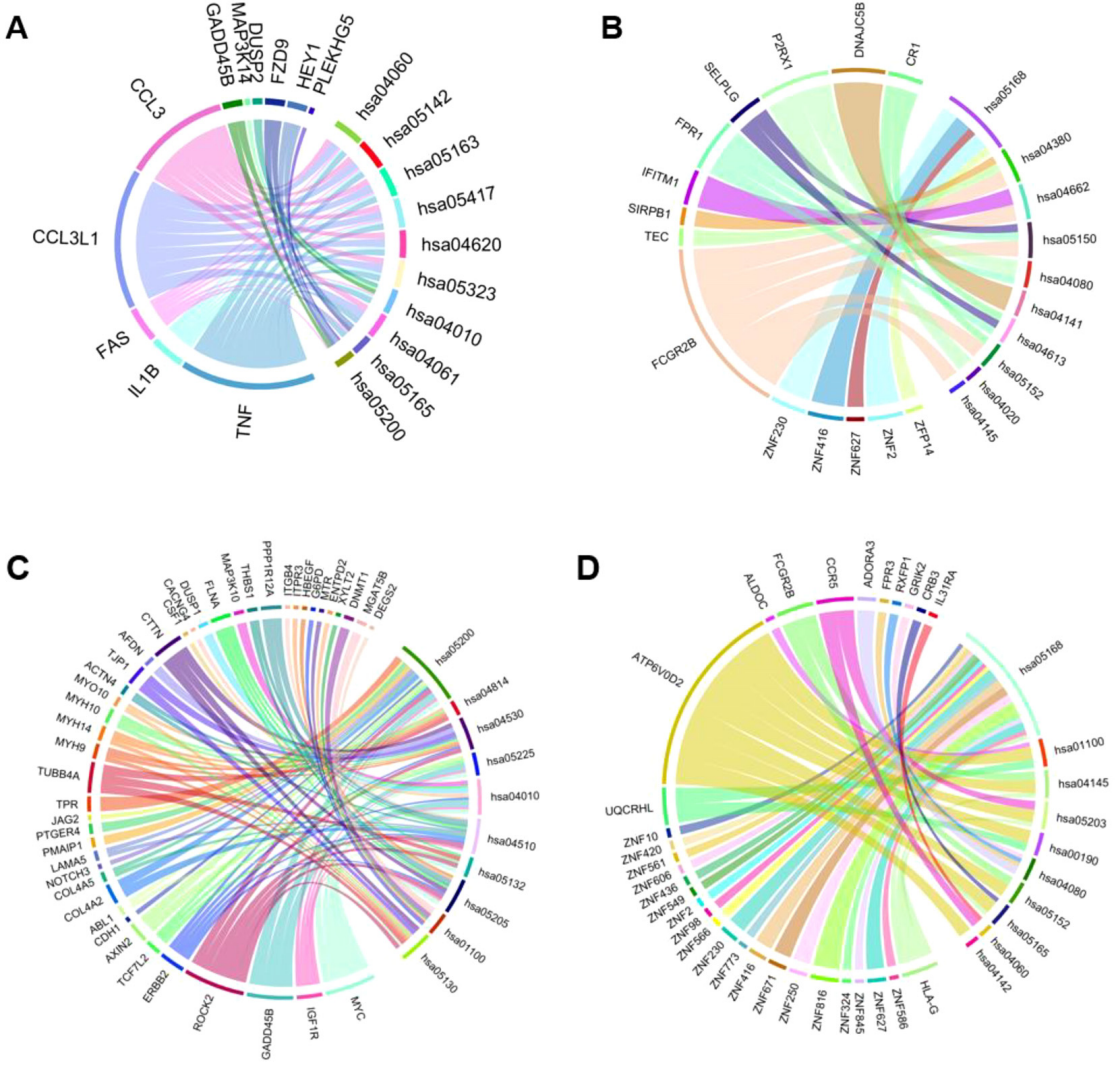
Chord diagrams depicting the associations between differentially expressed genes for cells and KEGG pathways (homo sapiens). **(A)** The top 10 KEGG pathways for upregulated genes in the apical compartment. **(B)** The top 10 KEGG pathways for downregulated genes in the apical compartment. **(C)** The top 10 KEGG pathways for upregulated genes in the basolateral compartment. **(D)** The top 10 KEGG pathways for downregulated genes in the basolateral compartment.

KEGG pathways map the network of gene products, focusing on disease pathways (38). We prepared chord diagrams representing the data obtained from KEGG pathway analysis, shown in **Figure 3**. These depict associations between differentially expressed genes for apical cells and basal cells and KEGG pathways. Upregulated and downregulated genes are shown, and each KEGG pathway is described (**Table 2**).

**Table 2:** Description of KEGG Pathways.

KEGG Pathway	Description
hsa04060	Cytokine-cytokine receptor interaction
hsa05142	Chagas disease
hsa05163	Human cytomegalovirus infection
hsa05417	Lipid and atherosclerosis
hsa04620	Toll-like receptor signaling pathway
hsa05323	Rheumatoid arthritis
hsa04010	MAPK signaling pathway
hsa04061	Viral protein interaction with cytokine and cytokine receptor
hsa05165	Human papillomavirus infection
hsa05200	Pathways in cancer
hsa05168	Herpes simplex virus 1 infection
hsa04380	Osteoclast differentiation
hsa04662	B cell receptor signaling pathway
hsa05150	*Staphylococcus aureus* infection
hsa04080	Neuroactive ligand-receptor interaction
hsa04141	Protein processing in endoplasmic reticulum
hsa04613	Neutrophil extracellular trap formation
hsa05152	Tuberculosis
hsa04020	Calcium signaling pathway
hsa04145	Phagosome
hsa05200	Pathways in cancer
hsa04814	Motor proteins
hsa04530	Tight junction
hsa05225	Hepatocellular carcinoma
hsa04010	MAPK signaling pathway
hsa04510	Focal adhesion
hsa05132	Salmonella infection
hsa05205	Proteoglycans in cancer
hsa01100	Metabolic pathways
hsa05130	Pathogenic Escherichia coli infection
hsa05168	Herpes simplex virus 1 infection
hsa01100	Metabolic pathways
hsa04145	Phagosome
hsa05203	Viral carcinogenesis
hsa00190	Oxidative phosphorylation
hsa04080	Neuroactive ligand-receptor interaction
hsa05165	Human papillomavirus infection
hsa04060	Cytokine-cytokine receptor interaction
hsa04142	Lysosome

The upregulated genes for the KEGG pathways to be *PLEKGH5*, *HEY1*, *FZD9*, *DUSP2*, *MAP3K14*, *GADD45B*, *CCL3*, *CCL3L1*, *FAS*, *IL1B*, *TNF* in the apical compartment, shown in **Figure 3**. The downregulated genes for the apical compartment are *CR1*, *DNAJC5B*, *P2RX1*, *SELPLG*, *FPR1*, *IFITM1*, *SIRPB1*, *TEC*, *FCGR2B*, *ZNF230*, *ZNF416*, *ZNF627*, *ZNF2*, *ZFP14*.

**Figure 3** shows the upregulated genes in the basal compartment to be *DEGS2*, *MGAT5B*, *DNMT1*, *XYLT2*, *ENTPD2*, *MTR*, *G6PD*, *HBEGF*, *ITPR3*, *ITGB4*, *PPP1R12A*, *THBS1*, *MAP3K10*, *FLNA*, *DUSP1*, *CACNG4*, *CSF1*, *CTTN*, *AFDN*, *TJP1*, *ACTN4*, *MYO10*, *MYH14*, *MYH9*, *TUBB4A*, *TPR*, *JAG2*, *PTGER4*, *PMAIP1*, *LAMA5*, *NOTCH3*, *COL4A5*, *COL4A2*, *ABL1*, *CDH1*, *AXIN2*, *TCF7L2*, *ERBB2*, *ROCK2*, *GADD45B*, *IGF1R*, *MYC*. The downregulated genes are *IL31RA*, *CRB3*, *GRIK2*, *RXFP1*, *FPR3*, *ADORA3*, *CCR5*, *FCGR2B*, *ALDOC*, *ATP6V0D2*, *UQCRHL*, *ZNF10*, *ZNF420*, *ZNF561*, *ZNF606*, *ZNF436*, *ZNF549*, *ZNF2*, *ZNF98*, *ZNF566*, *ZNF230*, *ZNF773*, *ZNF416*, *ZNF671*, *ZNF250*, *ZNF816*, *ZNF324*, *ZNF845*, *ZNF627*, *ZNF586*, *HLA-G*.

**Figure 4:**
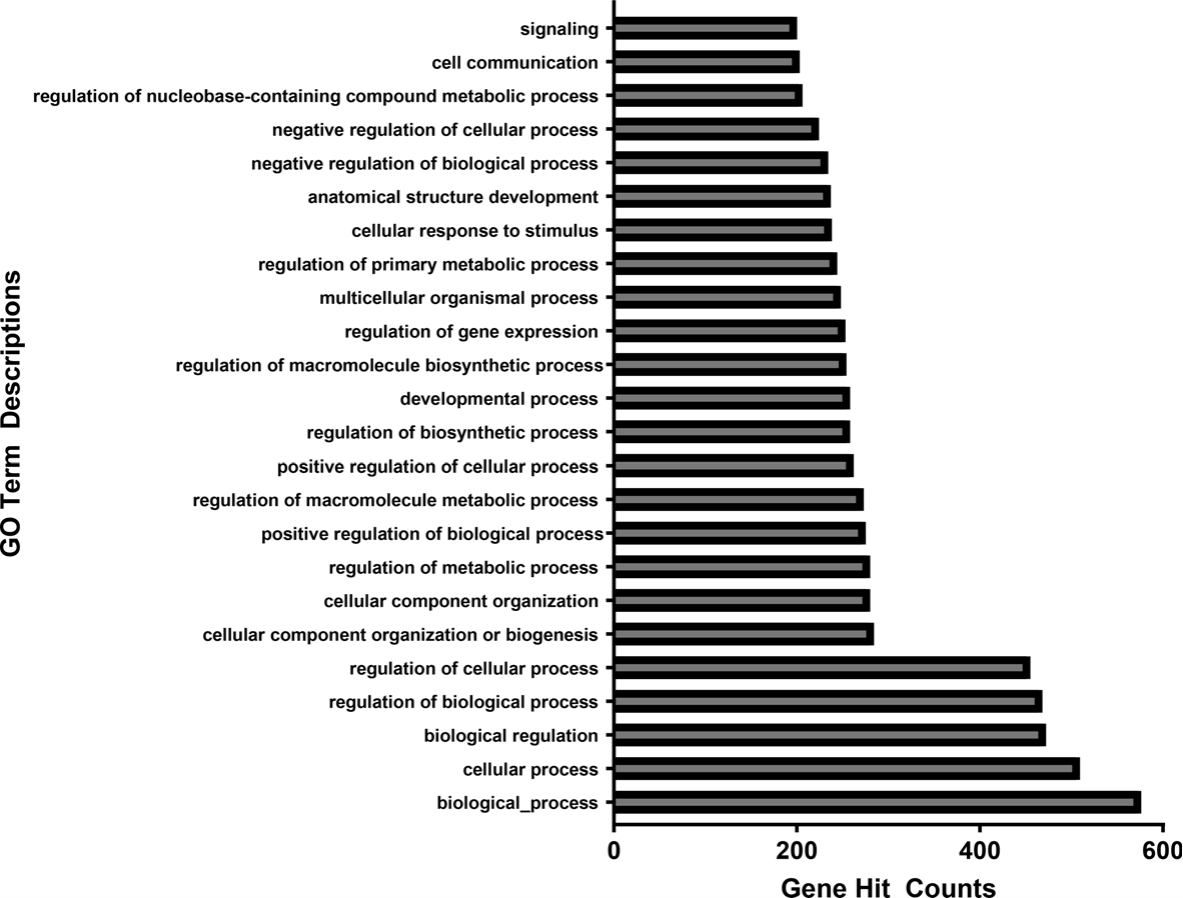
Associated gene counts for each GO term. Terms are arranged in order of total gene hit counts. 926 genes were uploaded into the GO database. From the 622 gene IDs that were accepted and processed, 570 had uniquely mapped IDs. Terms with less than 200 gene counts were omitted from this figure.

**Figure 5:**
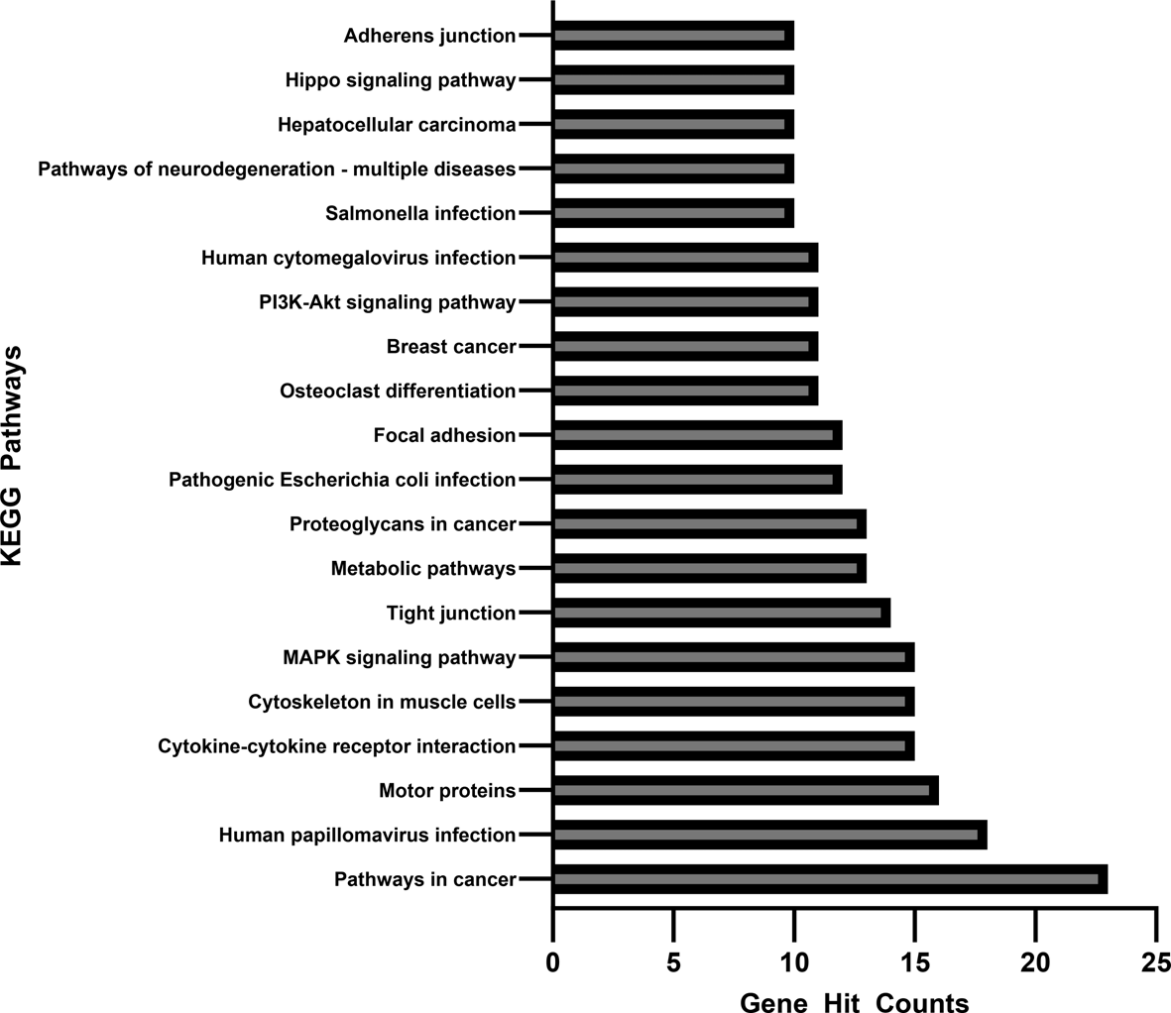
Associated gene counts for each KEGG pathway. Terms are arranged in order of total gene hit counts. 926 genes were uploaded into the KEGG database, and 368 were accepted and processed. Terms with less than 10 gene counts were omitted from this figure.

**Figures 4**, **5** each show the associated gene counts for each GO and KEGG term, respectively. These demonstrate the number of perturbed genes from the exposures related to each disease process or other perturbation.

**Figure 4** shows the number of genes associated with each GO term. Of the 926 genes uploaded into the GO database, 622 were accepted and able to be processed. 570 of those 622 genes had uniquely mapping gene IDs. GO terms with less than 200 associated gene counts were omitted from the figures. Each GO term in the final figure represented broad categories of cellular function, hence the high numbers of associated genes.

These differentially expressed genes were then input into the GO database to reveal common processes regulated by the affected genes with a hierarchical distance of 3 from the root biological process (2). For the upregulated genes, the most common GO terms were G protein-coupled receptor signaling pathway, negative regulation of cell population proliferation, cell differentiation, angiogenesis, humoral immune response, regulation of cell cycle, necroptotic signaling pathway, monoatomic ion transmembrane transport, osteoblast differentiation, vascular endothelial growth factor production, positive regulation of cell population proliferation, mitotic cell cycle, regulation of cell population proliferation, spermatogenesis, response to xenobiotic stimulus, microtubule-based movement, and actin cytoskeleton organization.

The downregulated genes had GO terms related to purinergic nucleotide & adenosine cell surface receptor signaling pathway, G protein-coupled receptor signaling pathway, innate immune response, negative regulation of cell population proliferation, response to a virus, positive regulation of phagocytosis, defense response, negative regulation of immune response, adaptive immune response, follicular dendritic cell activation, spermatogenesis, chemotaxis, negative regulation of immune response, the establishment of localization in cell, negative regulation of cytokine production, response to bacterium, and defense response.

The most common GO pathways for the apical compartment cells were chemokine receptor signaling, cell cycling, and humoral responses (upregulated) and G protein-coupled receptor signaling, innate and adaptive immune responses, and phagocytosis (downregulated). The pathways for the basal cells were cell differentiation, actin cytoskeleton organization, microtubule movement, response to xenobiotic stimulus (upregulated) and receptor signaling, chemotaxis, negative regulation of immune response, negative regulation of cytokine production, and defense response (downregulated).

**Figure 5** shows the number of genes associated with each KEGG pathway. Of the 926 genes uploaded into the KEGG database, 368 were accepted and able to be processed. Those with less than 10 associated gene counts were omitted from the figures.

The GO database detected a few more upregulated genes in the apical compartment than the KEGG database. However, in the basal compartment, the KEGG database found several more genes to be upregulated than the GO database did. In the same vein, the KEGG database found more genes to be downregulated in both the apical and basal compartments. The common genes found to be upregulated, in both the apical and basal compartment, between the GO and KEGG database include *ABL1*, *CCL3*, *CCL3L1*, *ERBB2*, *FAS*, *FLNA*, *FZD9*, *GADD458*, *IGF1R*, *IL1B*, *MAP3K14*, *MYC*, *PLEKHG5*, *PMAIP1*, *ROCK2*, *THBS1*, *TJP1*, and *TNF*. However, fewer common genes were downregulated in both the apical and basal compartments than upregulated. These include *ADORA3*, *ATP6V0D2*, *CCR5*, *CR1*, *FCGR2B*, *FPR1*, *HLA-G*, *ZNF2*, *ZNF98*.

The top 10 GO terms found to be upregulated in the apical compartment include GO:0071346, cellular response to type II interferon; GO:0030593, neutrophil chemotaxis; GO:0043065, positive regulation of apoptotic process; GO:0071407, cellular response to organic cyclic compound; GO:0048245, eosinophil chemotaxis; GO:0070098, chemokine-mediated signaling pathway; GO:0030198, extracellular matrix organization; GO:0043123, positive regulation of I-kappaB kinase/NF-kappaB signaling; GO:0051897, positive regulation of protein kinase B signaling; GO:0019722, calcium-mediated signaling.

The top 10 GO terms found to be downregulated in the apical compartment include GO:0006355, regulation of DNA-templated transcription; GO:0030183, B cell differentiation; GO:0043123, positive regulation of I-kappaB kinase/NF-kappaB signaling; GO:0043318, negative regulation of cytotoxic T cell degranulation; GO:0002430, complement receptor-mediated signaling pathway; GO:0050869, negative regulation of B cell activation; GO:0002865, negative regulation of acute inflammatory response to antigenic stimulus; GO:0002924, negative regulation of humoral immune response mediated by circulating immunoglobulin; GO:2001199, negative regulation of dendritic cell differentiation; GO:0002763, positive regulation of myeloid leukocyte differentiation.

The top 10 GO terms found to be upregulated in the basal compartment include GO:0043066, negative regulation of apoptotic process; GO:0006355, regulation of DNA-templated transcription; GO:0006468, protein phosphorylation; GO:0006338, chromatin remodeling; GO:0043065, positive regulation of apoptotic process; GO:0042981, regulation of apoptotic process; GO:0006897, endocytosis; GO:0006281, DNA repair; GO:0030198, extracellular matrix organization; GO:0006334, nucleosome assembly.

The top 10 GO terms found to be downregulated in the basal compartment include GO:0006355, regulation of DNA-templated transcription; GO:0035589, G protein-coupled purinergic nucleotide receptor signaling pathway; GO:0001973, G protein-coupled adenosine receptor signaling pathway; GO:0019722, calcium-mediated signaling; GO:2001199, negative regulation of dendritic cell differentiation; GO:0043552, positive regulation of phosphatidylinositol 3-kinase activity; GO:0070098, chemokine-mediated signaling pathway; GO:0051496, positive regulation of stress fiber assembly; GO:1902600, proton transmembrane transport; GO:0016241, regulation of macroautophagy.

The top 10 KEGG pathways for upregulated genes in the apical compartment include hsa04060, cytokine-cytokine receptor interaction; hsa05142, Chagas disease; hsa05163, human cytomegalovirus infection; hsa05417, lipid and atherosclerosis; hsa04620, toll-like receptor signaling pathway; hsa05323, rheumatoid arthritis; hsa04010, MAPK signaling pathway; hsa04061, viral protein interaction with cytokine and cytokine receptor; hsa05165, human papillomavirus infection; hsa05200, pathways in cancer.

The top 10 KEGG pathways for downregulated genes in the apical compartment include hsa05168, Herpes simplex virus 1 infection; hsa04380, osteoclast differentiation; hsa04662, B cell receptor signaling pathway; hsa05150, *Staphylococcus aureus* infection; hsa04080, neuroactive ligand-receptor interaction; hsa04141, protein processing in endoplasmic reticulum; hsa04613, neutrophil extracellular trap formation; hsa05152, tuberculosis; hsa04020, calcium signaling pathway; hsa04145, phagosome.

The top 10 KEGG pathways for upregulated genes in the basal compartment include hsa05200, pathways in cancer; hsa04814, motor proteins; hsa04530, tight junction; hsa05225, hepatocellular carcinoma; hsa04010, MAPK signaling pathway; hsa04510, focal adhesion; hsa05132, salmonella infection; hsa05205, proteoglycans in cancer; hsa01100, metabolic pathways; hsa05130, pathogenic *Escherichia coli* infection.

The top 10 KEGG pathways for downregulated genes in the basal compartment include hsa05168, Herpes simplex virus 1 infection; hsa01100, metabolic pathways; hsa04145, phagosome; hsa05203, viral carcinogenesis; hsa00190, oxidative phosphorylation; hsa04080, neuroactive ligand-receptor interaction; hsa05152, tuberculosis; hsa05165, human papillomavirus infection; hsa04060, cytokine-cytokine receptor interaction; hsa04142, lysosome.

**Figure 6:**
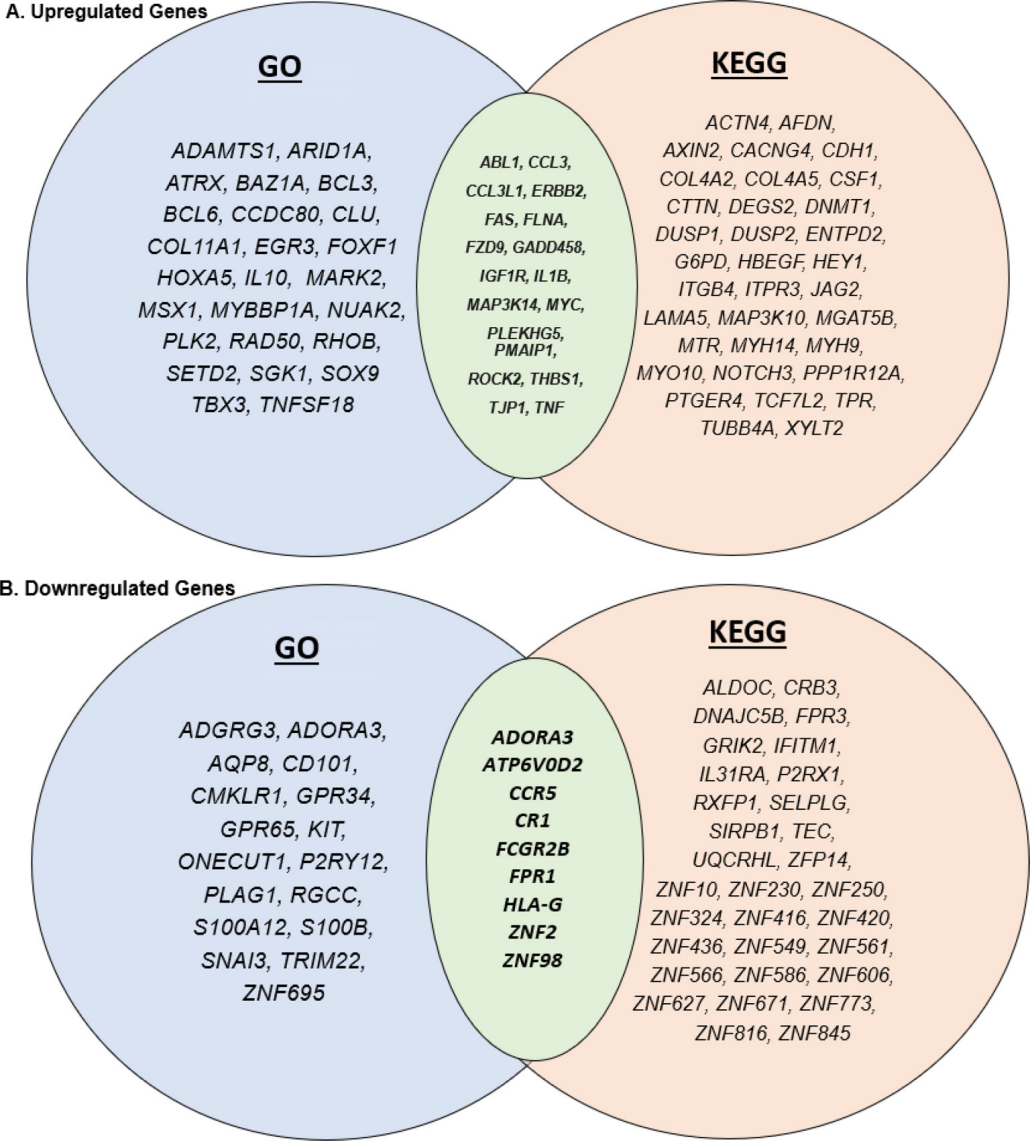
Venn diagrams depicting the similarities and differences between genes associated with GO and KEGG. **(A)** The common upregulated genes and **(B)** the common downregulated genes in KEGG and GO analyses.

By adding KEGG pathway analysis to this data set, this study can provide a more complete depiction of the genes affected by respiratory sensitizers (39–42). The genes that are discovered as upregulated by KEGG, but not by GO terms include *ACTN4*, *AFDN*, *AXIN2*, *CACNG4*, *CDH1*, *COL4A2*, *COL4A5*, *CSF1*, *CTTN*, *DEGS2*, *DNMT1*, *DUSP1*, *DUSP2*, *ENTPD2*, *G6PD*, *HBEGF*, *HEY1*, *ITGB4*, *ITPR3*, *JAG2*, *LAMA5*, *MAP3K10*, *MGAT5BI, MTR*, *MYH14*, *MYH9*, *MYO10*, *NOTCH3*, *PPP1R12A*, *PTGER4*, *TCF7L2*, *TPR*, *TUBB4A*, *XYLT2* (**Figure 6A**). The genes that are discovered as downregulated by KEGG but not by GO terms include *ALDOC*, *CRB3*, *DNAJC5B*, *FPR3*, *GRIK2*, *IFITM1*, *IL31RA*, *P2RX1*, *RXFP1*, *SELPLG*, *SIRPB1*, *TEC*, *UQCRHL*, *ZFP14*, *ZNF10*, *ZNF230*, *ZNF250*, *ZNF324*, *ZNF416*, *ZNF420*, *ZNF436*, Z*NF549*, *ZNF561*, *ZNF566*, *ZNF586*, *ZNF606*, *ZNF627*, *ZNF671*, *ZNF773*, *ZNF816*, *ZNF845* (**Figure 6B**). With KEGG pathway analysis, many genes that were perturbed when using the GO database were noticed as insignificant. This demonstrates the need to use multiple databases when performing pathway analysis to understand the effects of respiratory sensitizers or other irritants.

## Discussion

The GO database groups genes based on different subjects and qualities to develop a database that can annotate genes, gene products, and sequences (20). Specifically, GO seeks to integrate consistent gene product descriptors and standardize classifications for sequences and their features (29, 32). KEGG analysis aids in developing a list of related genes and assembling those genes into a proposed biological pathway. The resultant data can be used to probe protein interaction networks (43). These have shown to be beneficial when used together, such as reviewing chemicals to determine the difference between carcinogens and non-carcinogens and other toxic properties of chemicals that may prevent human exposure to harmful substances (38, 43–45).

While both databases are useful for pathway analysis, they are structured differently to be useful in different scenarios. While the KEGG database may be easier to understand due to its list-like structure, the GO database is used for a more complex view of the relationships between gene functions (30, 44). Therefore, choosing which databases to use is up to the researcher based on the study’s goals.

In a previous study, the GO database was used to evaluate the different biological processes affected by exposure to respiratory sensitizers (2). A variety of genes was found to be differentially expressed between cell culture models exposed to sensitizers versus non-sensitizers. The cells contained in the apical compartment of the model, the epithelial (A549) and macrophage (U937) cells, were compared to those cultured in the basal compartment, the dendritic cells (THP-1).

Gene expression is fundamentally related to protein expression; an excess or deficiency of proteins could lead to disease onset. Chen et al. (2015) (52) pre-classified drug-target interactions (DTI) and mapped a benchmark dataset consisting of 2,015 drugs that were assigned to nine biological endpoint categories (G protein-coupled receptors, cytokine receptors, nuclear receptors, ion channels, transporters, enzymes, kinases, antigens, and pathogens) using gene ontology and KEGG pathway enrichment analysis (38). The same research team, in 2016, connected the toxic properties of 171,266 chemicals retrieved from the Accelrys Toxicity Database (40). The categories of toxicity effects used in the analysis included acute toxicity, mutagenicity, tumorigenicity, skin and eye irritation, and reproductive effects.

Many other *in vitro* assays have previously focused on utilizing transcriptomic signatures to identify respiratory sensitizers. These assess the expression of genes related to the immune system, inflammatory cytokines, and associated cell signaling pathways (29, 44, 45). Other notable upregulated pathways for respiratory sensitizers included oxidative phosphorylation; ubiquinone metabolism; cytoplasmic/mitochondrial transport of proapoptotic proteins Bid, Bmf, and Bim; astrocyte differentiation; cell cycle regulation via Nek; ATP and ITP metabolism; dynein-dynactin motor complex in axonal transport; and insulin regulation of translation (29). Several of these pathways were also confirmed to be upregulated in this analysis. Many of these pathways are consistent when compared to the adverse outcome pathway for sensitization of the respiratory tract by low-molecular-weight chemicals presented by Sullivan et al. (42).

Though past research has investigated leveraging transcriptomics to identify respiratory sensitizers and distinguish sensitization types, most focus on a narrow set of endpoints: immune cell recruitment and inflammatory signaling pathways. Additionally, previous work has only considered upregulated genes, yet downregulated genes, which are considered here, also play a role in which proteins are present in the cellular environment. Finally, most previous studies used a monoculture cell model for data generation. This study expands this view of transcriptomics to a broader range of outcomes and uses data from a triculture model to create a more robust and physiologically relevant dataset. This work combines features from past studies with KEGG and GO data to build a more nuanced understanding of the genes and pathways affected by respiratory sensitization (46–54).

## Conclusions

The upregulated and downregulated genes identified through GO and KEGG analyses represent strong candidates for biomarker identification in respiratory sensitization. These pathways require further toxicological analyses. Developing a more comprehensive understanding of how the genes and pathways affect human health outcomes is essential for better understanding respiratory sensitization. Additionally, for future data analyses, utilizing the GO and KEGG analysis results is a powerful tool for identifying potential biomarkers related to adverse health conditions.

## Data Availability

The datasets presented in this study can be found in online repositories. The names of the repository/repositories and accession number(s) can be found below: PRJNA1141533 (BioProject).
